# HOME AND COMMUNITY HEALTHCARE FOR FRAIL OLDER ADULTS: A PATIENT, CAREGIVER, AND PRACTITIONER PERSPECTIVE

**DOI:** 10.1093/geroni/igz038.2546

**Published:** 2019-11-08

**Authors:** Andrew McDonald, Rowena Rizzotti, Joanna Rivera, Grace Park, Antonina Garm, Ryan D’Arcy, Xiaowei Song

**Affiliations:** 1 Health Research and Innovation, Surrey Memorial Hospital, Fraser Health, Surrey, British Columbia, Canada; 2 Health and Technology District, Surrey, British Columbia, Canada; 3 Health Research and Innovation, Surrey Memorial Hospital, Fraser Health Authority, Surrey, British Columbia, Canada; 4 Community Actions and Resources Empowering Seniors, Fraser Health Authority, Surrey, British Columbia, Canada; 5 Community Actions and Resources Empowering Seniors, Fraser Health Authority, British Columbia, Canada, Canada

## Abstract

Frailty and the decline in ability to maintain independent living may be forestalled through discussions with healthcare providers and seniors about managing health at home. In addition, the use of technology in supplementing doctors’ visits to assess frailty progression may be easily adopted by some but not others. We conducted this qualitative study to elucidate the context in which seniors access care at home and current barriers to independent living, from the perspectives of both seniors and practitioners. Pre-approved discussion questions were administered to two audio-recorded focus group sessions of 14 participants. The first group were community-dwelling older adults and informal caregivers, while the second consisted of healthcare practitioners. Group members were sampled to include a range of health backgrounds and levels of technological expertise. Thematic analysis with NVivo Software was used to parse out key discussion topics from the audio transcripts. The caregiver/patient group emphasized the stigma of frailty and age-related isolation, desiring transparency and advocacy from care teams. Practitioners/researchers discussed the importance of a holistic biopsychosocial approach to frailty management and the need for standardized frailty measurement. Patients/caregivers used health-tracking devices at home and were more optimistic about telehealth/video-conferencing than practitioners. Awareness of contextual factors surrounding “aging in place” and what aspects of care are valued by patients and practitioners is key to advancing home health and paving the way for new evidence-based services in the home.

